# Experiences of Pathology Course among Hospital Management Graduates

**DOI:** 10.3390/healthcare9030347

**Published:** 2021-03-18

**Authors:** Jung Hee Park, Woo Sok Han, Jinkyung Kim, Hyunjung Lee

**Affiliations:** 1 Department of Emergency Medical Service, Konyang University, Daejeon 35365, Korea; jhpug@konyang.ac.kr; 2 Department of Hospital Management, Konyang University, Daejeon 35365, Korea; wshan@konyang.ac.kr (W.S.H.); jkim@konyang.ac.kr (J.K.); 3 College of Nursing, Konyang University, Daejeon 35365, Korea

**Keywords:** hospital management, pathology, qualitative study

## Abstract

The purpose of this study was to explore hospital management graduates’ experience in pathology courses. Data were gathered through four focus group interviews by 16 hospital management graduates who attended pathology courses. Data were collected from June to August, 2020. Conventional content analysis was used for data analysis. Six categories were extracted that described hospital management graduates’ experience in pathology courses, as follows: “Suggestions for the curriculum,” “Students’ preference for pathology professor,” “Demands for various teaching methods,” “Broad and difficult class content,” “Recognition of pathology courses during college years,” and “The importance of studying the pathology course realized after graduation.” The findings suggest that it is important to identify hospital management graduates’ perspectives to improve pathology curriculum in the educational process. Additionally, it is necessary to continuously connect educational and practical environments for the effective management of pathology courses.

## 1. Introduction

The department of hospital management encourages and trains professionals in the field of health administration by teaching theories and practices that are essential for the effective management of different health care organizations, with an emphasis on basic medical education [1]. Students majoring in hospital management build their careers in the health and medical industry, and most of the graduates are employed in medical institutions. Different departments associated with health administration, such as the department of public health, department of health care management, department of health administration, department of health policy, and department of hospital management, follow a similar curriculum. In South Korea, since the 1980s, health administration departments have distributed their curriculum over four-year bachelor courses at colleges, demonstrating how four-year-course college departments are gradually subdividing and specializing in accordance with the current trends and the social demand [2].

Currently, with the advancement of information technology, life science, and genetics, the medical industry is rapidly changing [3]. Thus, practitioners in the field of health administration are expected to show multiple competencies such as basic medical knowledge, knowledge about the systems used in the medical field, operation, and management skills [1]. The primary duties assigned to health administration majors in medical institutions include medical records and insurance reviews alongside computerized data management, hospital administration, planning and public relations, human resources, supplies, and the general administration of the hospital. As such, it is necessary to cultivate professionalism that can be used in overall work related to hospital management. Previous study findings revealed that the level of satisfaction among college students majoring in health administration had a positive correlation with career preparation and decisions [2,4,5], and the level of satisfaction with the curriculum of their major had a significant effect on becoming qualified and employed in the respective fields [6]. Therefore, colleges with four-year-courses offering majors in hospital management include classes on basic medical fields such as anatomy, pathology, and physiology in their curriculum; this aids in strengthening the competencies of students majoring in hospital management [7,8]. Additionally, the department of health administration belongs to the colleges of health or medical science, with the department of hospital management. Furthermore, it was found that basic medical education, including pathology, was chiefly taught by professors majoring in basic medicine or nursing [1,4]. Previous studies on basic medicine have largely focused on students majoring in nursing or medical engineering [9]; however, there have been very few studies on the effectiveness of basic medicine as a subject for departments related to health administration. This gap may have occurred due to the lack of importance given to basic medicine compared to other subjects. However, this subject is central because students from the department of hospital management are often employed in fields relevant to medicine; therefore, they need to have knowledge about diseases to enable seamless communication with other medical workers and to understand patients. In addition, hospital management graduates can be put into overall hospital management-related tasks. Since medical knowledge is required to support the work of healthcare workers, the awareness of the importance of pathology is increasing in clinical practice [1,3]. However, the hospital management department belongs to the college of health sciences or medical sciences, which focus on hospital management, health insurance claims, administration, and statistics rather than professional knowledge in medicine. To that end, both professors and students have difficulties in teaching and studying pre-clinical medical classes such as pathology and anatomy [6]. The results of overall course evaluations, which are customarily done at the university level, have only shown a formal quantitative score and have limitations in understanding students’ needs. Thus, more in-depth exploration was required. Moreover, it is important to understand the students’ educational needs and implement effective instructional strategies to promote competencies among learners [6,9]. Therefore, with the rapid development in the multidisciplinary field of medicine, it is essential to examine the pathology course curriculum from the perspective of students majoring in hospital management.

The ADDIE (Analysis, Design, Development, Implementation, and Evaluation) model, which structures the teaching–learning planning in different stages (analysis, design, development, implementation, and evaluation) [10], enables educators to create programs using a systematic approach designed to meet learner’s needs. Thus, this study aimed to thoroughly understand the experience of students attending pathology courses who are majoring in hospital management; this study conducted focus group interviews and analyzed the results to serve as basic data for the effective management of pathology courses by reflecting on the perspectives of the graduates.

## 2. Materials and Methods

### 2.1. Study Design

This phenomenological qualitative study aimed to obtain insights into the essence of hospital management graduates’ experiences by vividly describing the experiences of a pathology course in at Konyang University.

### 2.2. Participants

Participants were recruited in this study by posting a recruitment notice on an online portal with an active audience of hospital administration graduates. Graduates who agreed to participate in the research further introduced the researchers to their colleagues. The inclusion criteria for the participants of this study were: (1) those who had majored in hospital administration, (2) those who had attended pathology courses when enrolled in hospital administration, (3) those who had graduated in the last five years, and (4) those who were employed in fields relevant to their major. A total of 16 individuals met all the inclusion criteria and provided informed consent to participate in the study. It is recommended to conduct three or more focus groups with four to six participants in each to extract and obtain reliable data [11,12]. Therefore, participants were divided into four groups as per their workplace (medical records office group, hospital administration group, hospital office group of the same university hospital, and insurance company group) to ensure the homogeneity of participants and free flowing discussions in each group (Table 1).

### 2.3. Interviews

The questions were formed in accordance with the format recommendations of Krueger and Casey; our interview questionnaire comprised an opening question, introductory questions, transitional questions, main questions, and a closing question in an open-ended question format [11,12]. The questions were reviewed by a qualitative research expert before the final completion to ensure that their content did not deviate from the research investigation. The overall purpose of the interview was to obtain an answer to “How was your experience of taking pathology courses while in school?” Other questions are shown in Table 2.

### 2.4. Data Collection

Qualitative research uses the principle of “appropriacy” for sampling; therefore, it is important to select participants who can provide the most relevant information about the research topic and objective [13]. Focus group interviews, in particular, enable a broader understanding of the research topic since intensive conversations among participants with common characteristics generate detailed research material [11,12]. Thus, four interviewees were assigned to each group to facilitate effective conversation management and enable participants to freely express their feelings.

Focus groups were led by skilled moderators experienced in qualitative research and familiar with the scope of this research. The first author attended most of the focus groups. If this was not possible for logistic reasons, another co-author familiar with the interview protocol took over. The data collection period was from 25 July to 15 August 2020, and the interviews were conducted in a quiet and comfortable location as per the participants’ convenience and availability. There were two times of interview at each group, approximately 1 h and 1 h and 20 min long for each session, and participants were notified of the topic and questions of the study prior to the interview. Data collection was finished after second interview when no new information was generated from the interviews; therefore, data from 4 previously selected groups with 16 participants were finally included.

### 2.5. Data Analysis

The data transcribed by two assistant researchers were analyzed based on the consensual qualitative research (CQR) developed by Hill et al. [14]. The analysis followed an in-depth process. First, the researchers repeatedly listened to the recorded interviews, accurately transcribed the interviews, and then reviewed the participant protocols several times to identify and understand the underlying emotions throughout the interviews. Second, the researchers recorded the initial feelings experienced by the participants, the first thoughts that occurred to them, and the initial analysis of the data, and then they generated codes for their statements. Third, the researchers conducted a detailed discussion to record sub-themes for core factors of the responses; additionally, the extracted sub-themes were combined to form clusters of themes with similar contents, and these theme clusters were grouped into domains. Throughout the entire data analysis process, the researchers met several times and continuously contacted each other to compare, discuss, and agree on the data analyzed by each of them. In addition, to evaluate the appropriateness of the extracted codes, sub-themes, and theme clusters, the process was repeated to reconfirm and revise the original statements. Researchers proceeded with the analysis immediately after the data were collected, and repeatedly interviewed the participants until there was no new content in the following interviews. Once the statements, concepts, and themes started to appear repeatedly in the data analysis, the process was deemed to have reached theoretical saturation. The first and co-authors separately coded and analyzed the data, and the corresponding author supervised this process.

Data were sent to the participants via e-mail after analysis and reporting to identify their accuracy with the participants’ experiences and to improve the credibility of the study; furthermore, participant feedback was obtained for this purpose. In order to maintain neutrality, the researchers conducted an extensive literature review on the research subject, and all judgments were reserved throughout the process to avoid researchers’ prejudice or bias as much as possible.

### 2.6. Pre-Understanding

The researchers of this study were professors who have experience in teaching pathology courses, in addition to clinical and hospital administration experience at university hospitals. They have continuously participated in seminars to conduct qualitative studies, explored and discussed qualitative research methods, and developed their capabilities for qualitative research. Moreover, a mentor professor provided general guidance on qualitative research performance over the course of this study. In addition, the corresponding author of this study studied qualitative research in graduate school, conducted qualitative research for the last five years, and published it in academic journals.

### 2.7. Ethical Considerations

This study was approved by the Institutional Review Board of Konyang University, which the authors were affiliated prior to data collection (IRB No 2020-096-01). Participants were informed about withdrawing their participation at any time during the study with no disadvantages, and their consent was obtained to record the interview. The audio files and transcripts of the recordings were coded to prevent the identification of the participants’ identities, and separate unique numbers were assigned. Documents were stored in a document storage box with a lock, and they were discarded using a shredder after the completion of the study.

## 3. Results

A total of 232 meaningful statements were extracted from the original data provided by the 16 participants. We extracted two broad content areas: (1) regarding course management and (2) regarding the learners. These areas were further classified into six theme clusters and 12 themes (Table 3).

### 3.1. Area: Course Management

The course management area included four theme clusters: “Suggestions for the curriculum,” “Students’ preference for pathology professor,” “Demands for various teaching methods,” and “Broad and difficult content.” Participants provided their opinions based on their personal experience of the pathology course.

#### 3.1.1. Theme Cluster: Suggestions for the Curriculum

This theme cluster was composed of three themes: “the need to assign relevant courses,” “the necessity to understand the educational level and needs of students,” and “the desire to learn before or after the hospital placement.” The participants shared their subjective experiences and existing difficulties with the curriculum.

##### Theme: The Need to Assign Relevant Courses

Participants frequently experienced the need to study the relevant material prior to attending these classes due to the use of technical terminology and unfamiliar diseases in the pathology course. It was also considered necessary to learn by sequentially assigning the pathology course and subjects relevant to pathology courses to help with education in pathology, which was considered difficult to learn.

*“I think it would be better to study (pathology) together with a field or part such as pharmacology or anatomical physiology, and …such subjects will be more beneficial, so I think it will be more beneficial if pathology was studied together during the semester that pharmacology or anatomical physiology is studied.”*

(H3)

##### Theme: The Necessity to Understand the Educational Level and Needs of Students

Pathology is an unfamiliar area of study for these students and provides basic medical knowledge. Therefore, participants hoped to learn smoothly without difficulties by being taught at an eye-level; they wanted the professors to consider the students’ educational level while teaching. In addition, participants suggested that understanding the educational needs of the students will help in establishing an in-depth course for students who wish to learn in-depth contents in accordance with the diverse interests and concerns of the pathology course.

*“Because the professor teaches from a completely different background of knowledge from us, when we are trying to accept the content, um… I thought it would have been good if the professor could explain it more easily and extensively.”*

(P4)

*“For students who want to learn pathology even during their undergraduate years because they find it fun, this would be an opportunity for them to choose... so, if there is an opportunity to receive the education then I think the students who want to study further could do so and use it in their future somehow.”*

(L1)

##### Theme: Desire to Learn before or after the Clinical Practice

The participants’ opinions about the study period of the pathology course varied. Several participants had hoped to learn pathology during the second or third year in order to ensure practical application during their clinical practice in hospitals. Participants generally agreed that pathology courses had a positive influence on clinical practice at hospitals.

*“I think the semester students can usually focus is around the second year. I don’t think there will be a lot to do for employment or to attain other qualifications, so I think it would be good to learn intensively before the clinical practice.”*

(H3)

*“Now that I have completed the clinical practice, I think learning in the first semester of the third year and then immediately following it with clinical practice would be a good idea.”*

(P1)

#### 3.1.2. Theme Cluster: The Pathology Professor the Students Want

This theme cluster consisted of two themes: “the professor with extensive hospital clinical experience” and “the professor who interacts with students and teaches in a way that students can easily learn.” Participants presented their opinions about the characteristics they desired in their professors based on their experiences and impressions formed during classes.

##### Theme: Professor with Extensive Hospital Clinical Experience

In accordance with the unfamiliarity and difficulty of the pathology course, students desired a professor who could help them easily understand the topic. Professors, who taught using their real-life clinical experiences and case studies, engaged the students’ interest in a fun and memorable way during the pathology course.

*“The vivid experiences of the clinical field encountered through the professors enriched the pathology course memories.”*

(K4)

*“We are now graduating and moving forward because the professor gave lectures to us while working in the hospital field and…professors are normally employed in hospitals or insurance companies, which enables us to hear a lot about the direct experiences that they had while working, so …that helped a lot.”*

(P4)

##### Theme: Professor Who Interacts with Students and Teaches in a Way That Students Can Easily Learn

Since pathology was a difficult subject, learners reflected their positive experience with professors based on the highest learning effect. Participants positively evaluated those professors who usually attempted to interact with students, and their efforts subsequently led to high learning outcomes.

*“The professor asked the students if anyone had ever suffered a disease, and how and where they were sick. The professor asked a lot of questions like this, so I think that helped a lot learn intensively before the pathology was conducted pleasant classes, which… was a good method.”*

(H1)

#### 3.1.3. Theme Cluster: Demands for Various Teaching Methods

This theme cluster consisted of two themes: “memorable case-based role-play classes” and “boring lecture-style classes.” The participants desired learner-centered teaching methods based on their previous learning experience.

##### Theme: Memorable Case-Based Role-Play Classes

In terms of learning methods, participants wanted learner-centered class plans conducted through various mediums that were easy to approach and participate in.

*“It is good when they give case scenarios and give some time to think about the examinations or diagnosis…”*.

(L2)

*“It wasn’t just lectures, but we were grouped together we were given a disease each and did something like a role play. So, I think it was good that I was able to learn and understand more specifically about the disease while directly participating in the role play, in addition to the theoretical part of the disease…”*.

(P1)

*“We did something like a role-play in the last class of pathology. Each group was assigned the name of a disease and played the roles of a doctor, a patient, and a nurse for such situation. It was followed by an evaluation, and I wondered why we were doing such a thing back then. Looking back now, I think that was what helped me.”*

(L4)

##### Theme: Boring Lecture-Style Classes

In general, there were many negative evaluations for lecture-style classes (professor-centered).

*“When I listened to the lecture, had to wrote it down without thinking, so I became much less attentive and …just listening to lectures was not suitable for me”*.

(L2)

*“I wasn’t able to concentrate due to um…incomprehensive terminology and boring lecture…”*.

(K2)

#### 3.1.4. Theme Cluster: Broad and Difficult Class Content

This theme cluster consisted of two themes: “excessive amount of learning content” and “difficulties in concentrating on basic medical content.” The participants had various content requirements based on their current employment, and they wanted practical contents that could be applied to their job duties.

##### Theme: Excessive amount of Learning Content

Participants’ primary memory of the pathology classes was the difficulty faced due to the unfamiliarity and large proportion of the curriculum. The excessiveness of the content was especially higher for liberal arts students who were completely unaware of basic medicine.

*“There were too many disease to learn and memorize.”*

(H1)

*“It was difficult because the amount of study was too much compared to other subjects.”*

(K4)

##### Theme: Difficulties in Concentrating on Basic Medical Content

Participants complained about difficulties in concentrating during the classes due to the use of unfamiliar and technical pathological terminology of pathology, which is starkly different from those used in other hospital administration courses, and the rapid progression into specialized medical content.

*“Due to the nature of the pathology, there is a lot of medical knowledge and a lot of information to be delivered…Personally, it was difficult to understand because there was a lot of information being given at once.”*

(P4)

*“Incomprehensible terminology gave me hard time…”*.

(P1)

### 3.2. Area: The Learners

This area consisted of two theme clusters: “recognition of pathology courses during college years” and “the importance of pathology courses participants learned after graduation.” Study participants suggested ideas to help the future students gain a positive experience, based on their personal experiences. This was classified as the learners’ area.

#### 3.2.1. Theme Cluster: Recognition of Pathology Courses during College Years

This theme cluster consisted of two themes: “lack of motivation to learn” and “subjects requiring memorization that participants wanted to give up.” Participants emphasized the necessity and importance of learning pathology and the role of learners who are the focal point of education in accordance with their real-life experiences.

##### Theme: Lack of Motivation to Learn

The participants believed that there was a lack of awareness about the effect of pathology courses on their future practice.

*“Since we do not do practical work but rather do administrative work, I think I attended the pathology classes with an underlying opinion that pathology is not important.”*

(K4)

##### Theme: The Memorization Subjects That Participants Wanted to Give Up

Participants revealed that a lack of motivation for learning pathology, the excessive amount of studying, and the realization that pathology requires memorization negatively affected the learning outcomes.

*“Students give up because there is so much to learn … hard to understand and probably because they do not want to memorize.”*

(L3)

*“All I can recall is the memorization techniques.”*

(P2)

#### 3.2.2. Theme Cluster: The Importance of Studying the Pathology Course Realized after Graduation

This theme cluster consisted of two themes: “coming to know that pathology is an important course that is helpful in clinical practice” and “helpful in obtaining a major-related qualification.”

##### Theme: Coming to Know That Pathology Is an Important Course That Is Helpful in Clinical Practice

The participants emphasized the importance and usefulness of the pathology course based on their positive experiences during their employment practice; although they did not recognize this importance while attending the classes.

*“It helped me greatly to understand which areas are diseases with high severity and to understand whether patients have high severity when reading the list.”*

(H3)

*“I think once you go to work after understanding the subject called pathology, you can communicate more comfortably with people in the medical department, nurses, or even with the patients you directly encounter.”*

(K4)

*“It is content you need to know after you get a job and results will change according to how you study so I hope students study hard and learn a lot.”*

(L2)

##### Theme: Helpful in Obtaining a Major-Related Qualification

The participants in this study vividly remembered their experiences while preparing for employment after graduation. Participants conveyed the pre-requisite role of the pathology course in obtaining further certification related to their major, such as that of a health information manager. Additionally, they acknowledged that studying the course provided practical guidance for obtaining the certification.

*“There are a lot of places that require a certificate than you would expect...They prefer people with a certificate...and it’s a very good certificate to have...I think studying pathology will be absolutely indispensable to get a certificate such as health information manager certificate in the future.”*

(K1)

*“I think it was helpful when I was taking the medical recorder’s license exam. It really helped my studies when I looked through the materials I had studied before.”*

(L3)

## 4. Discussion

This paper presents a qualitative study that aimed to understand the underlying experience of studying in a pathology course among hospital management graduates, and six theme clusters were subsequently extracted. An unexpected question from the participants during the interview was when they asked for an explanation of what a pathology course entails. This was surprising because all the participants had opted into this course and had only graduated within the last five years. Therefore, this indicates the relatively low importance attached to basic courses in hospital management majors, as administration and management courses are considered more essential. However, a general understanding of disease is becoming necessary with the increased engagement in medicine-related duties. Fortunately, the participants began to comprehensively reminisce about the overall experience of the course management and learning throughout the rest of the interview.

Pathology involves the in-depth study of disease mechanisms; H3 of the hospital office team expressed the desire to sequentially study relevant courses such as medical terminology, anatomy, physiology, and clinical practice. H3 also suggested the need for conducted pathology courses, before or after hospital placements, at the medical institutions in consideration with the educational level and needs of the students. Previous studies with students of health and medical department-related subjects have revealed that satisfaction with the major subject was high if high satisfaction was experienced with the curriculum and field placement [4,5,6,15]. This demonstrates that a systematically designed curriculum has a significant influence on overall course satisfaction. Similarly, other participants of the current study also wanted the pathology course to be taught before or after hospital placements. Therefore, it is necessary to conduct detailed discussions about the completion period of the course while designing the curriculum in order to increase student satisfaction with basic courses among hospital management graduates. These changes in curriculum design will aid the clinical placement of students, which can be an important turning point for those studying subjects relevant to health and medical departments; moreover, it will help in satisfying their intellectual curiosity related to their major and in fostering their potential [15]. Furthermore, due to the adaptive and dynamic nature of the curriculum, the pathology content may be added or reduced according to the educational needs of learners [4,5,6,15]; therefore, it is necessary to assess student satisfaction with the pathology course for those who are currently enrolled.

The second theme cluster in course management was concerned with student preferences for the professor of the course. Students preferred pathology professors who had clinical experience in hospitals and taught students in an interactive way that facilitated easy learning. Since professors enjoy a high degree of autonomy in class planning and evaluation, their competency acts as a crucial factor in determining the quality of their classes [16]. Students preferred professors with practical clinical experience who facilitate easy learning for students using real-life clinical cases during the pathology lectures. The results of a previous study reported that highly prioritized competencies in college professors are “systematic compositions” and “the explanation capacity” [17]. The current study findings were consistent with the results of this previous study. Thus, professors must seek techniques to systematically organize the contents of the pathology curriculum to easily explain the contents for students studying hospital administration while emphasizing important core contents.

Participants also expressed difficulty to motivate themselves to learn pathology during their college years, as the subject dealt with the mechanism of diseases. Therefore, professors with specialized knowledge and experience should use clinical case studies to make pathology classes less technical and more practical.

These requests were categorized into the third theme cluster of course management concerning the demands for various teaching methods, which included classes using audio–visual materials and case-based, role-playing techniques as the preferred lesson methods in order to tackle the difficulties expressed by the participants to engage in learning with lecture-based lessons due to boredom. The characteristics of excellent instructors are well-organized class content, clear delivery, and harmonious interactions with students [18]. Rote learning with professor-centered education to deliver extensive amounts of knowledge was the method preferred in the past when knowledge itself was the resource. However, modern day students require learner-centered classes (e.g., problem-based and team-based learning) to acquire and apply knowledge by participating in various learning activities based on student–professor interactions [9]. Due to the gap in previous research on the management of basic courses such as pathology for hospital management students, this study reviewed the available literature on the educational needs of nursing students, which is another health-related department. The reviewed literature revealed that nursing students also preferred performance-oriented education that enabled learning through direct participation and experiential learning, which was similar to those studying hospital administration [19]. Role-playing education, which participants positively recalled, involves the process of autonomously constructing specific scenarios, planning roles, and promoting the understanding of diseases; it can be an effective learning method that improves problem-solving and self-directed learning skills A previous study identified “media and technology utilization ” as a component of an appealing class [20]; therefore, professors must seek methods to appropriately use relevant audio–visual materials to promote the interests and understanding in learners regarding unfamiliar disease mechanisms and terminology.

In the fourth theme cluster about the broad and difficult class content, the participants’ complaints regarding the excessive amount of learning material and the basic medical contents that were difficult to concentrate on were recorded. Participants expressed the following concerns: (1) difficulty due to technical terminologies, (2) unfamiliarity with the content, (3) limitations due to the excessive amount of study material, (4) treating the course as a memorization subject, and (5) giving up learning altogether.

This is connected with the recognition of pathology courses during college years, which was the first theme cluster in the learner’s area. Participants suggested that they were indifferent to the pathology course during their school years due to a lack of motivation to learn and because it was a subject that they wanted to give up studying due to the extensive amount of memorization involved. However, participants independently confirmed the importance of basic knowledge about diseases during their employment after graduation; they eventually realized the importance of the pathology course. Such factors are important for motivating students studying hospital management to learn pathology. As the learning and academic achievement increases, the purpose of taking a class becomes clearer for a learner [21]. These experiences of graduates can have a positive effect on inducing learning motivation for students who are currently enrolled.

A lack of effort or awareness in a learner who is the focus of education can prevent correct learning and lead to the abandonment of an course altogether [21]. In other words, a learner’s proactive learning attitude is a determining factor in achieving learning outcomes.

Participants suggested that their experience of taking a pathology course as a student was crucial for reviewing and determining medical records at work in the medical institutions and insurance companies where they currently work. However, they expressed regret over not recognizing this importance during college. These findings demonstrate highly meaningful statements for professors teaching pathology in the field of education. Since the career paths of students studying hospital management are associated with medical institutions, it is imperative to improve their ability to seamlessly communicate with other health care workers and to understand their patients. Therefore, specific goals need to be determined for students studying hospital management to achieve the learning outcomes of the pathology course. Furthermore, there is also an urgency to seek solutions that consider the educational needs and the academic achievement of students while planning pathology classes to implement education at the eye-level for students who are studying hospital management.

## 5. Conclusions

This study divided the experience of the pathology course among graduates of hospital management into the areas of: (1) course management and (2) learners. The graduates commonly and consensually expressed a lack of motivation to study pathology and difficulties in concentrating on the course content due to the excessive amount of study material. However, graduates appeared to have recognized the importance of the pathology course after graduation during their employment in fields relevant to their major.

There are limitations in generalizing these results because this study conducted focus group interviews with the graduates of hospital management from the same local university. However, this study is significant because it has provided a deeper understanding of the experience of studying the pathology course in hospital management students amidst an insufficiency of relevant literature.

Based on the results of this study, the following recommendations are proposed: (1) the current management of basic courses, including pathology, in the department of hospital management must be evaluated and recognized; (2) pathology course curriculum should facilitate practical learning for effective the study management of pathological courses, which could be facilitated through collaborations with the employment centers where students can work after their graduation; and (3) learning contents should be constructed based on major diseases to enhance learning motivation. The findings of this study will be helpful for developing pathology course for hospital management students.

## Figures and Tables

**Table 1:** General characteristics of study participants (N = 16).

Participant		Age (Years)	Sex	Degree	Working Experience
**L**	1	25	F	Bachelor	3 years
	2	25	F	Master	3 years
	3	26	M	Bachelor	2 years
	4	26	F	Bachelor	4 years
P	1	27	F	Bachelor	4 years
	2	26	M	Bachelor	1 years
	3	26	F	Bachelor	2 years
	4	27	F	Bachelor	1 years
K	1	26	F	Master	3 years
	2	24	F	Bachelor	1 years
	3	24	F	Bachelor	1 years
	4	23	F	Bachelor	1 years
H	1	23	F	Bachelor	1 years
	2	23	F	Bachelor	1 years
	3	27	M	Bachelor	2 years
	4	24	F	Bachelor	1 years

L: medical records office team; P: Insurance company team; K: hospital administration team; H: hospital office team.

**Table 2:** Questions for this study.

Opening Question	Please Share Your Experience of the Pathology Courses You Attended While You Were in College.
Introductory questions	Please share the satisfying experiences you had during the course.
	Please share the unsatisfying experiences you had during the course.
Transitional questions	Please share about any difficulties you had during the course.
	Do you think taking pathology classes helped you with your current job?
	Please share your overall experience at your professor’s lectures.
	Please share your overall experience at your pathology courses curriculum
Closing question	Please share anything you may want to mention.

**Table 3:** Area, theme clusters, and themes of hospital management graduates’ pathology course experiences.

Area	Theme Clusters	Themes
Course management	Suggestions for the curriculum	The need to assign relevant courses
		The necessity to understand the educational level and needs of students
		Desire to learn before or after the clinical practice
	Students’ preference for pathology professor	Professor with extensive hospital clinical experience
		Professor who interacts with students and teaches in a way that students can easily learn
	Demands for various teaching methods	Memorable case-based, role-play classes
		Boring lecture-style classes
	Broad and difficult class content	Excessive amount of learning content
		Difficulties in concentrating on basic medical content
The learners	Recognition of pathology courses during college years	Lack of motivation to learn
		The memorization subject participants wanted to give up
	The importance of studying the pathology course realized after graduation	Coming to know that pathology is an important course that is helpful in clinical practice
		Helpful in obtaining a major-related qualification

## Data Availability

The data presented in this study are available on request from the corresponding author. The data are not publicly available due to data restriction policies.
